# Bioengineered coagulation factor VIII enables long-term correction of murine hemophilia A following liver-directed adeno-associated viral vector delivery

**DOI:** 10.1038/mtm.2014.36

**Published:** 2014-08-06

**Authors:** Harrison C Brown, J Fraser Wright, Shangzhen Zhou, Allison M Lytle, Jordan E Shields, H Trent Spencer, Christopher B Doering

**Affiliations:** 1 Graduate Program in Molecular and Systems Pharmacology, Laney Graduate School, Emory University, Atlanta, Georgia, USA; 2 Center for Cellular and Molecular Therapeutics, The Children’s Hospital of Philadelphia, Philadelphia, Pennsylvania, USA; 3 Perelman School of Medicine, University of Pennsylvania, Philadelphia, Pennsylvania, USA; 4 Department of Pediatrics, Aflac Cancer and Blood Disorders Center, Emory University School of Medicine, Atlanta, Georgia, USA

## Abstract

Clinical data support the feasibility and safety of adeno-associated viral (AAV) vectors in gene therapy applications. Despite several clinical trials of AAV-based gene transfer for hemophilia B, a unique set of obstacles impede the development of a similar approach for hemophilia A. These include (i) the size of the factor VIII (fVIII) transgene, (ii) humoral immune responses to fVIII, (iii) inefficient biosynthesis of human fVIII, and (iv) AAV vector immunity. Through bioengineering approaches, a novel fVIII molecule, designated ET3, was developed and shown to improve biosynthetic efficiency 10- to 100-fold. In this study, the utility of ET3 was assessed in the context of liver-directed, AAV-mediated gene transfer into hemophilia A mice. Due to the large size of the expression cassette, AAV-ET3 genomes packaged into viral particles as partial genome fragments. Despite this potential limitation, a single peripheral vein administration of AAV-ET3 into immune-competent hemophilia A mice resulted in correction of the fVIII deficiency at lower vector doses than previously reported for similarly oversized AAV-fVIII vectors. Therefore, ET3 appears to improve vector potency and mitigate at least one of the critical barriers to AAV-based clinical gene therapy for hemophilia A.

## Introduction

Hemophilia A is an X-linked congenital bleeding disorder characterized by a deficiency in functional coagulation factor VIII (fVIII) in the blood compartment. Recently, clinical advancements have been made using recombinant adeno-associated virus (rAAV)-based gene transfer for hemophilia B.^1^ However, a unique set of obstacles impede the development of a similar approach for the related and more common bleeding disorder hemophilia A. These obstacles include (i) inefficient biosynthesis of human fVIII (hfVIII) compared to other plasma proteins such as factor IX,^2^ (ii) limited packaging capacity of rAAV (4.7 kb)^3,4^ which is exceeded by all fVIII encoding rAAV genomes since the B domain deleted fVIII transgene alone is greater than 4.4 kb, (iii) humoral immune responses to circulating fVIII,^5^ and (iv) capsid-mediated cytotoxicity of the virus itself, for which clinical data suggests occurs at doses as low as 2e12 vector particles (vp)/kg for AAV serotypes 2 and 8.^6^

FVIII is a large glycoprotein containing the domain structure A1-A2-B-activation peptide(ap)-A3-C1-C2. Human fVIII is produced at levels 3 orders of magnitude lower than other similarly sized secreted glycoproteins both *in vivo* and *in vitro*. This low secretory rate has been spatially and temporally linked to inefficient posttranslational trafficking from the endoplasmic reticulum to the Golgi.^2,7–11^ The primary determinants of this biosynthetic limitation are specific amino acid sequences within the A1 and A3 domains of the molecule itself that cannot be overcome by standard transgene expression technologies such as more efficient DNA regulatory elements (*e.g.*, promoters/enhancers), transgene copy number, targeted integration at transcriptionally active sites, and codon optimization. Instead, bioengineering of the fVIII molecule itself appears to be required to overcome inefficient secretion. We have developed such a bioengineered fVIII molecule (herein designated ET3 and previously referred to as HP47) using knowledge gained from the characterization of B-domain deleted (BDD) recombinant porcine fVIII (rpfVIII).^12,13^ Both rpfVIII and ET3 are secreted 10- to 100-fold more efficiently than other fVIII constructs through diminished interactions with endoplasmic reticulum resident chaperones and attenuated induction of the unfolded protein response.^11–14^ The enhanced secretory capacity of ET3 is enabled through the substitution of “high expression” rpfVIII sequences residing in the A1 and *ap-*A3 domains into recombinant human fVIII (rhfVIII). Despite accounting for only 9% overall sequence modification, these limited amino acid substitutions have been shown to be necessary and sufficient to confer 10- to 100-fold improved biosynthesis.^13^ In addition to the A1 and *ap*-A3 domain substitutions, the BDD hfVIII protein, designated HSQ, substitutes the 14 amino acid human-derived SQ linker sequence for the B-domain, whereas ET3 utilizes a24 amino acid porcine sequence-derived OL linker sequence, which has been described previously and shown to confer no additional expression/biosynthesis advantage over the SQ linker.^12^

In this study, proof of concept of a liver-directed rAAV vector encoding ET3 to confer long-term expression of therapeutic levels of fVIII in immune-competent hemophilia A mice was investigated. The vector design was based on a rAAV vector previously shown to effectively deliver and express the human coagulation factor IX (fIX) transgene.^15^ This current vector, designated rAAV-HCR-ET3, has a total genome size of 5.9 kb, which is 125% of the 4.7 kb endogenous rAAV genome and therefore is oversized. Recently published data from Samulski and colleagues suggest that oversized AAV genomes are packaged into preformed viral capsids as incomplete single stranded (ss) DNA fragments with either strand polarity (+ or −) and containing overlapping portions of the transgene that are heterogeneously truncated during the packaging process. Although functional intracellular reassembly of these transgene fragments presumably through Watson-Crick base pairing was shown to occur, transgene product expression levels were demonstrated to be 25- to 37-fold lower than that attained from smaller, non-fragmented transgenes.^3,4,16,17^ The standard mechanism of AAV reassembly and second strand synthesis has been reviewed previously.^18^ Given the limitations of generating complete rAAV vectors for large transgenes such as fVIII, understanding the mechanisms and efficiencies of oversized rAAV genome packaging and transgene cassette reassembly are critical to the development of next generation of rAAV vectors.

## Results

### Viral vector construction and *in vitro* comparison of BDD hfVIII and ET3 expression

**Figure 1:**
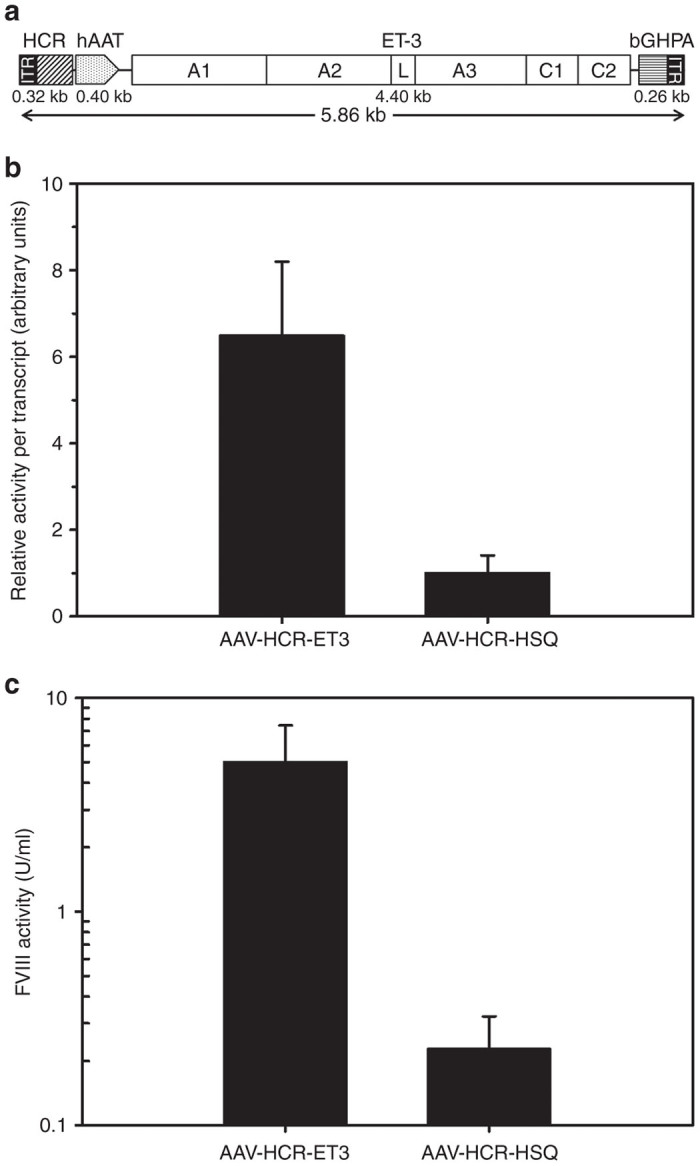
Viral vector design and *in vitro* expression. The 5.86 kb rAAV-HCR-ET3 genome encodes the high expression bioengineered fVIII molecule ET3, which consists of porcine fVIII sequences in the A1 and *ap-*A3 domains and human sequence in the A2, C1, and C2 domains. The ET3 transgene is under the control of a liver-specific hAAT promoter/ApoEHCR enhancer sequence, and termination is governed by a bovine growth hormone poly adenylation signal (bGHPA). (**a**) The genome is flanked by AAV2 ITRs on both the 5′ and 3′ ends. fVIII activity was measured in conditioned medium of HepG2 cells that were transiently transfected with rAAV-HCR viral expression vectors encoding ET3 or the non-bioengineered BDD hfVIII construct HSQ. fVIII mRNA levels were measured by quantitative reverse transcription PCR. (**b**) For comparison of biosynthetic efficiency, the ratio of fVIII activity production to fVIII mRNA transcripts per cell is displayed. (**c**) Plasma fVIII activity was measured in hemophilia A mice that were hydrodynamically injected with rAAV-HCR viral expression plasmid encoding ET3 or HSQ. Baseline fVIII levels were determined to be below the limit of detection for all mice (data not shown). All error bars show one sample standard deviation, *N* = 3 for *in vitro* studies and 3–4 for *in vivo* studies.

The rAAV vector design was based on constructs previously used to express the human coagulation factor IX transgene from liver tissue.^15^ The ET3 transgene, which consists of human fVIII sequences in the A2, C1, and C2 domains and porcine fVIII sequences in the A1 and *ap-*A3 domains, or alternatively, a BDD hfVIII transgene with a 14 amino acid linker (SQ), designated HSQ, was cloned into an AAV expression cassette controlled by a liver-specific ApoEhepatic control region (HCR)/human alpha-1 antitrypsin (hAAT) enhancer/promoter and flanked by AAV2 inverted terminal repeats (ITRs) (Figure 1a). Complete amino acid alignment of HSQ and ET3 amino acid sequences reveals 91 percent identity (Supplementary Figure S2). We previously have shown that the increased expression of ET3 is conferred through enhanced posttranslational processing of the nascent fVIII peptide.^13^ To determine if improved biosynthetic efficiency was retained by the AAV-HCR-ET3 expression cassette, an *in vitro* transfection experiment utilizing the human hepatocellular carcinoma HepG2 cell line was performed. AAV-HCR-ET3 and AAV-HCR-HSQ expression plasmids were transiently transfected into HepG2 cells for assessment of fVIII transcript levels and secreted fVIII activity. Although cells transfected with AAV-HCR-ET3 plasmid contained greater numbers of fVIII mRNA transcripts per cell than those transfected with AAV-HCR-HSQ (850 ± 39 versus 284 ± 69), this 3-fold differential in mRNA level could not account for the >20-fold differential in fVIII activity observed in the conditioned medium (0.70 ± 0.24 units (U)/ml for ET3, and 0.034 ± 0.01 U/ml for HSQ). Thus, AAV-HCR-ET3 transfected HepG2 cells demonstrated sevenfold higher levels of fVIII production per mRNA transcript than the AAV-HCR-HSQ transfected cells suggesting that post mRNA biosynthetic efficiency of ET3 expression, presumably endoplasmic reticulum to golgi transit, is the primary determinant of high level expression in the context of AAV based liver-directed expression (Figure 1b). However, we cannot rule out that increased transcriptional efficiency or mRNA stability may further contribute to the enhanced expression of ET3 compared to HSQ. To further examine the finding of enhanced expression of ET3, an *in vivo* comparison of the two vector-transgene designs by hydrodynamic injection of the expression plasmids was performed. In this experimental system, again the AAV-HCR-ET3 expression plasmid conferred 20-fold higher plasma levels of fVIII activity than AAV-HCR-HSQ expression plasmid further supporting the claim of enhanced production of ET3 compared to HSQ (Figure 1c, Supplementary Table S3).

### rAAV vector production and characterization

AAV particles encoding the HCR-ET3 transgene cassette were generated by transient transfection of HEK293 cells and subsequent purification of the vector particles from supernatants and cell lysates as previously described.^19^ RAAV-HCR-ET3 was designed with a vector genome of 5.9 kb from end to end inclusive of both ITRs, which exceeds the endogenous rAAV genome size by 25%. Despite its oversized design, production of ~1.2e13 total rAAV-HCR-ET3 vp at titers of 5.3e12 vp per ml was achieved.

**Figure 2:**
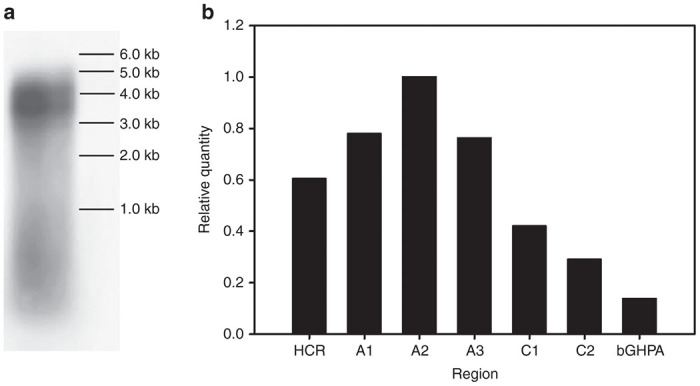
Molecular assembly of rAAV-HCR-ET3 vector particles. (**a**) SsDNA from rAAV-HCR-ET3 viral particles were purified, subjected to alkaline gel electrophoresis, and detected by Southern blot. (**b**) The molecular composition of the packaged viral genomes was determined by quantitative PCR directed against specific regions of the vector genome and normalized to the quantity of the A2 region.

To assess the effect of the oversized genome on rAAV packaging, viral ssDNA obtained from cesium chloride gradient purified rAAV-HCR-ET3 was subjected to alkaline gel electrophoresis followed by Southern blot analysis using probes directed to the A2 and C2 domain sequences of fVIII and the bovine growth hormone (BGH) polyadenylation signal sequence (Figure 2a). The rAAV-HCR-ET3 vector preparation did not contain detectable genetic material at the position expected for full-length genomes (5.9 kb). Rather, a heterogeneous smear of viral ssDNA approaching 5.0 kb was observed suggesting that the majority of viral genomic DNA was packaged as truncated fragments. It has been suggested previously that oversized transgenes, such as that encoded by rAAV-HCR-ET3, may extend beyond the capsid, exposing free 5′ ends of ssDNA on the outside of the viral particles. A comparison of Southern blot analysis of viral particles treated with DNAse prior to disruption of the viral capsid to those that were not DNAse treated failed to detect a difference in genome size, suggesting that vector genomic ssDNA does not extend beyond the viral capsid (data not shown).

The composition of the truncated rAAV-HCR-ET3 genome fragments was assessed by qPCR using primer sets directed against different genome regions spanning from ITR to ITR (Figure 2b). Although this analysis could not distinguish strand polarity (+ or −), it did show that sequences corresponding to the A2 domain of fVIII, which are located near the center of the designed viral genome, were the most prevalent. Comparatively, the terminal sequences, both encompassing either the promoter or poly A signal sequence, were up to 7.3-fold less prevalent than the central A2 sequences. This result is consistent with the current theory that oversized AAV viral genomes are packaged from one of the 3′ ITRs of either polarity and truncated prematurely before reaching the other ITR.

### *In vivo* expression of fVIII rAAV-HCR-ET3

**Figure 3:**
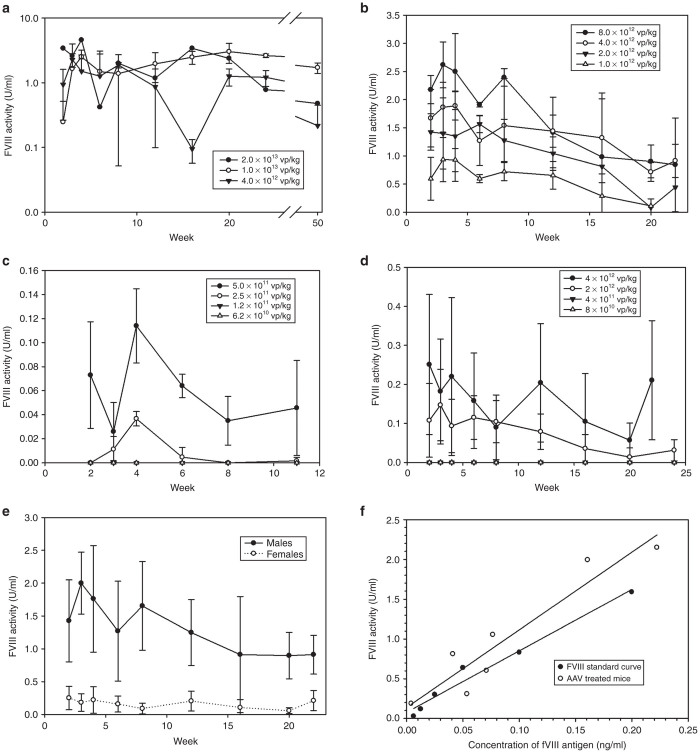
Dose finding of rAAV-HCR-ET3 in a murine model of hemophilia A. Dose finding studies were performed in male and female immune-competent hemophilia A mice. fVIII activity is presented from mice that did not form neutralizing antibodies. In male mice, (**a**) a long term, high dose study; (**b)** a medium duration, mid-dose extension study; and (**c**) a short term, minimum effective dose study were performed. In female mice, (**d**) a shorter duration dose finding study was performed. (**e**) A comparison between male and female mice receiving 4e12 vg/kg shows the intersex difference in fVIII levels. Baseline determination of fVIII activity in all mice tested was below the limit of detection of the assay (data not shown). *N* = 2–4 for all doses, error bars show one sample standard deviation. To determine the specific activity of *in vivo* rAAV expressed ET3, a panel of plasmas from rAAV-HCR-ET3 treated mice (open circles) were assayed for ET3 antigen level by ELISA and fVIII activity by chromogenic substrate activity assay. (**f**) The values obtained for each plasma were compared to a standard curve generated using highly purified recombinant ET3 diluted in hemophilia A mouse plasma (closed circles).

A dose finding study was performed to determine the ability of rAAV-HCR-ET3 to provide therapeutic levels of circulating fVIII activity *in vivo*. Immune-competent 8–12 week old male mice were administered a single peripheral vein injection of the vector at doses ranging from 6.2e10 to 2.0e13 vp/kg. A long term (50 weeks) study was performed on a first cohort of mice receiving high vector doses (4e12–2e13 vp/kg), while follow-up on the second cohort that received lower doses (1e12–8e12 vp/kg) was shorter (24 weeks). Finally, as the lower dose limit was not attained in the second cohort, a third cohort of mice was dosed with rAAV-HCR-ET3 at even lower levels (6.2e10–5e11 vp/kg) and was followed out to 11 weeks after AAV injection. Starting 2 weeks after vector administration, circulating plasma was assayed for fVIII activity at scheduled time points. Male mice receiving rAAV-HCR-ET3 showed dose responsive increases in circulating fVIII activity (Figure 3a–c). Supraphysiologic fVIII levels, at over >3 U/ml, were achieved at the dose of 1e13 vp/kg. For reference, the fVIII activity level observed in pooled normal human plasma is defined as 1 U/ml, while clinical hemophilia A disease classifications are severe (<1%), moderate (1–5%), and mild (5–40%) normal fVIII activity. Correction to curative levels (>0.4 U/ml or 40% normal human fVIII activity level) was achieved at doses as low as 1.0e12 vp/kg, and partial correction (>0.05 U/ml) was seen at doses as low as 5e11 vp/kg. fVIII expression was maintained throughout the duration of the experiment in all but cohorts receiving the lowest doses of vector administered and therefore defining a minimal effective dose. However, there was a trend toward activity loss over the duration of the experiment, which is consistent with gradual liver tissue turnover and loss of the episomal AAV genomes.

### Formation of anti-fVIII inhibitors

**Figure 4:**
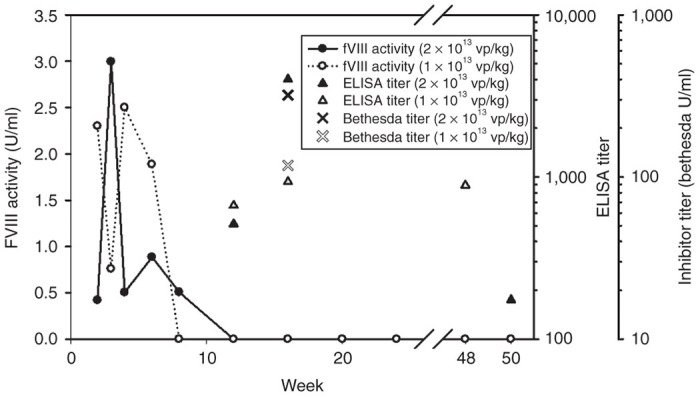
Formation of fVIII inhibitors following rAAV-HCR-ET3 administration. Two mice that received one of the high doses of rAAV-HCR-ET3 (2e13 vp/kg and 1e13 vp/kg) experienced sudden and persistent loss of fVIII activity at between 8 and 12 weeks after vector administration. Anti-ET3 ELISA assay confirmed these mice had developed IgG antibodies to fVIII, which persisted for the duration of the study. A Bethesda assay was performed at week 16 to confirm the presence of neutralizing antibodies against fVIII.

Two mice that received high doses of rAAV-HCR-ET3 (2e13 and 1e13 vp/kg) displayed sudden and sustained losses of circulating fVIII activity at weeks 8 and 12, respectively (Figure 4). Prior to the disappearance of plasma fVIII activity, both mice were expressing ET3 at supraphysiologic levels (>250% of normal). As repeated intravenous administration of recombinant fVIII (rfVIII) to naive hemophilia A mice is known to be immunogenic, all mice were tested for inhibitory antibodies to fVIII by ELISA. In the two mice that lost circulating fVIII activity, positive anti-fVIII ELISA titers of 512 and 668 were observed at week 12 after rAAV-HCR-ET3 infusion suggesting that that these animals had mounted neutralizing humoral immune responses against the transgene product, which persisted for the duration of the study. The antibodies were confirmed to be inhibitory by Bethesda assay at week 16, which showed inhibitor titers of 320 and 118 Bethesda units (BU) per ml. Development of inhibitors, as detected either by loss of fVIII activity or ELISA titer, was not observed in any of the other experimental mice throughout the course of the long term follow-up (data not shown).

### Expression of fVIII rAAV-HCR-ET3 in female mice

Previously, it has been reported that rAAV has reduced ability to mediate liver gene transfer in female mice.^20^ Therefore, a limited dose finding study for rAAV-HCR-ET3 was performed in 8–12 week old immune-competent female mice. At doses ranging from 8e10 to 4e12 vp/kg, female mice showed an attenuated response to rAAV-HCR-ET3 gene transfer, with doses as low as 2e12 vp/kg resulting in partial correction of fVIII activity, which was maintained for the duration of the study (Figure 3d). No female mice developed neutralizing antibodies to fVIII at the vector doses tested. With respect to the results obtained in male hemophilia A mice, circulating fVIII activity levels in female mice were approximately eightfold lower at all doses tested, which is consistent with previous reports (Figure 3e).

### Molecular composition of rAAV-derived fVIII

Molecular studies of the rAAV-HCR-ET3 vector suggests that the majority of viral genomes package as sub-5.0 kb fragments, which lack either the necessary 5′ transcriptional control region or 3′ fVIII coding sequence and poly(A) signal necessary to confer production of full-length fVIII protein. To confirm the expression of complete ET3 mRNA sequences in mice treated with vector, RNA was extracted from the livers of treated and untreated mice and was subjected to reverse transcription PCR analysis for the presence of ET3 C2 domain RNA sequence, a region spanning position 5036 to 5205 at the 3′ end of the transgene predicted to be truncated during the packaging process (Supplementary Figure S3). Truncation at the 5′ end of the expression cassette during packaging of the minus strand genomes, which includes the promoter region, would preclude transcription of ET3 mRNA, whereas truncation as the 3′ end of the expression cassette, which includes the termination codon and the polyadenylation sequence would not generate a template for the PCR reaction. Thus, experimental detection of this mRNA sequence supports the theory of AAV genome reassembly initiated by overlapping ssDNA fragments and resulting in functional expression cassettes capable being transcribed into full-length fVIII mRNA molecules.

While this analysis supports the presence of full length fVIII mRNA transcripts, it does not exclude the possibility that this outcome occurs at low frequency and full length fVIII transcripts may represent only a fraction of the AAV derived mRNA transcripts present in treated animals. Furthermore, the presence of incomplete mRNA transcripts could lead to the production of a heterogeneous population of dysfunctional fVIII molecules. To interrogate treated animals for the presence of dysfunctional fVIII, we measured the *in vivo* specific activity of rAAV-derived fVIII in rAAV-HCR-ET3 vector-treated hemophilia A mice. Antigen concentration was measured using a capture ELISA designed to detect the N terminal heavy chain of fVIII and quantified using a standard curve of highly purified recombinant ET3 diluted into hemophilia A mouse plasma. The specific activities of ET3 within the AAV treated animal plasma samples were found to be similar to that of recombinant ET3 spiked into murine hemophilia A plasma indicating that the majority of ET3 antigen present in these animals is functional and is not indicative of heterogeneous ET3 biosynthesis.

### Viral genome copy number in transduced livers

**Figure 5:**
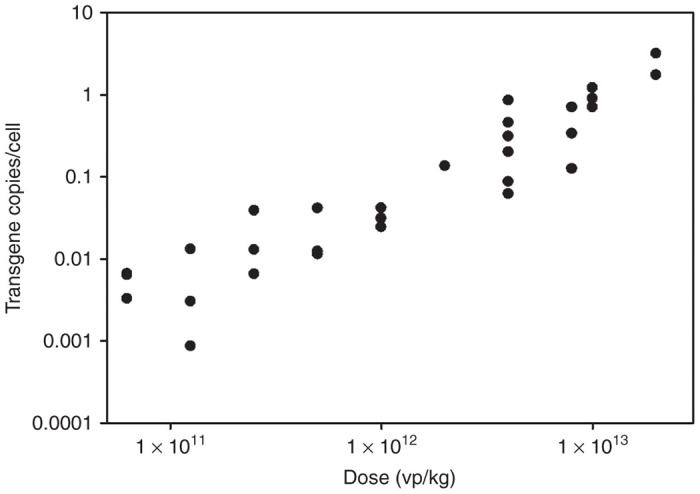
*In vivo* viral genome copy number. Quantitative PCR was used to determine the number of viral genomes in transduced livers. Primers directed against the fVIII A2 domain were used to determine the number of viral genome copies of this domain per cell for all mice receiving rAAV-HCR-ET3.

QPCR analysis of viral genome target sequences within the centrally located A2 domain of fVIII was performed using DNA extracted from livers of all transduced male mice (Figure 5). RAAV-HCR-ET3 proviral genomes containing the A2 domain sequence persisted as long as 1 year after treatment. There were approximately two viral transgenes per cell in mice receiving 2e13 vp/kg and a dose responsive decrease in transgene number was observed at the lower doses with mice receiving the lowest doses (6.4e10 vp/kg) approaching the limit of detection.

### Vector efficacy

**Figure 6:**
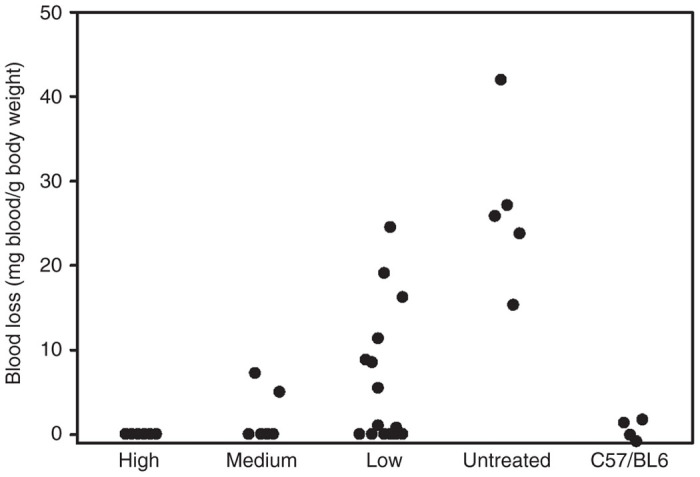
Phenotypic correction of the bleeding diathesis. Hemophilia A mice treated with rAAV-HCR-ET3 were subjected to a tail transection bleeding assay to test for phenotypic correction. For clarity, mice are grouped by vector dose: High (8e12–2e13 vp/kg), medium (2e12–8e12 vp/kg) low (6.3e10–1e12 vp/kg) and no treatment control.

Correction of the hemophilia A bleeding phenotype was assessed by tail transection bleeding assay on animals that received varying doses of rAAV-HCR-ET3 (Figure 6). Overall, mice that received rAAV-HCR-ET3 treatment demonstrated less blood loss than saline-treated control mice. Specifically, all mice that received one of the high vector doses (8e12–2e13 vp/kg) showed no detectable blood loss while mice that received medium (1e12–4e12 vp/kg) or low (6.2e10–5.0e11 vp/kg) doses demonstrated dose responsive decreases in blood loss that, both as individual groups and as a whole, were significantly less than saline treated controls (*P* < 0.005, Mann–Whitney *U*-test).

## Discussion

The favorable safety profile, ease of delivery, durable expression and lack of innate immune stimulation, contribute to the attractiveness of rAAV as a vector for clinical gene therapy of hemophilia A. However, large transgene size, potential humoral immunogenicity and poor biosynthetic efficiency of human fVIII present as significant obstacles hindering clinical translation. This point is supported by the observation that five clinical trials of AAV-factor IX vectors for the treatment of hemophilia B have been conducted or currently are in progress, while no clinical trials of rAAV-fVIII vectors in persons with hemophilia A have been initiated.

To overcome these obstacles, several advancements in AAV-fVIII vector design have been reported. For example, Lu *et al*. described an rAAV-fVIII construct incorporating a β-actin promoter with a cytomegalovirus enhancer and a bovine growth hormone poly(A) sequence that expressed fVIII at levels three- to fivefold higher than a comparable AAV construct utilizing a mini-transthyretin promoter with a synthetic poly(A) sequence.^21^ The use of these large regulatory control elements resulted in a final transgene size of 5.8 kb, which is similar to rAAV-HCR-ET3 and substantially oversized. Despite having no detectable vector genomes larger than 5.0 kb, the optimized vector developed by Lu *et al*. provided correction of fVIII levels in hemophilia A mice at a dose of 2e11 vector genomes (vg)/mouse, or ~8e12 vg/kg. Additionally, Arruda and colleagues recently described a minimally bioengineered hfVIII transgene delivered via an oversized (5.5 kb) rAAV vector achieving circulating fVIII levels up to 60% of normal at a dose of 8e12 vg/kg.^22^ This bioengineering approach incorporates a single amino acid substitution which eliminates a PACE/furin cleavage site within human fVIII to mimic what occurs naturally in the B domain of canine fVIII. This substitution conferred a 2.5-fold increase in secretion of single chain fVIII, as well as greater specific activity and improved hemostatic potency. Lastly, Nathwani and colleagues have developed a novel rAAV-fVIII vector that is reduced in size to 5.2 kb through promoter and polyadenylation sequence engineering and encodes a codon-optimized BDD hfVIII transgene that also incorporates a bioengineered linker containing additional N-glycan sequence motifs.^23^ The combination of these engineered elements resulted in a vector that mediated 10- to 20-fold higher expression of fVIII compared to the non-engineered vector. However, the exact mechanism(s) responsible for the improved expression were not determined.

In this study, we sought to enhance the potency of rAAV-fVIII vectors through the integration of a fVIII molecule that has been bioengineered for more efficient cellular secretion, which is known to be the rate-limiting step in recombinant fVIII biosynthesis and presumably also exists as a barrier in gene therapy settings. This novel approach may be complimentary to the advancements described above by others in the field and combinations of the technologies, such as (i) reduction in AAV genome size through the use of smaller gene regulation elements like the ones described by Nathwani and colleagues or (ii) the incorporation of the canine B-domain substitution described by Arruda and colleagues or (iii) the insertion of N-glycan motifs into the fVIII B-domain linker sequence, which could potentially result in additive or synergistic improvements in vector performance. This study demonstrates that rAAV-HCR-ET3 can effectively achieve curative levels of fVIII activity *in vivo* using a minimal dose of 1e12 vp/kg, which represents an eightfold reduction in vector dose needed to achieve curative levels of circulating fVIII compared to the results obtained by the groups of Lu and Arruda discussed above, and doses comparable to that achieved with the vector developed by Nathwani and colleagues despite the substantially oversized nature of rAAV-HCR-ET3. As production of sustained therapeutic levels of fVIII remains a critical barrier to clinical gene therapy of hemophilia A and no recent transformational advances have been made in the areas of vector manufacturing or potency, transgene engineering approaches, such as ET3, may provide the key innovation necessary to achieve clinical success. Furthermore, the reduced viral vector dose needed to achieve curative levels of fVIII represents a significant improvement in safety over other AAV-fVIII constructs through reduction in the viral antigen load delivered to the recipient, which previous and ongoing clinical trials have shown to initiate a cytotoxic immune response against transduced cells at vector doses as low as 2e12 vp/kg.^6^

Our laboratory has spent considerable effort studying the differential efficiency of fVIII biosynthesis, as well as other biochemical properties, of orthologous fVIII molecules. The knowledge gained has been applied to the bioengineering of enhanced fVIII molecules such as ET3/HP47 with markedly improved biosynthetic efficiency and increased stability following thrombin activation.^13^ Previously, we described the improved performance of ET3 and other similar high expression fVIII sequence element containing constructs over standard human BBD fVIII constructs in heterologous recombinant protein production systems as well as gene therapy applications involving γ-retroviral and lentiviral vector gene transfer into hematopoietic stem cells.^12–14,24–26^ However, this study is the first report utilizing the high expression sequence elements of porcine fVIII to improve fVIII levels in a liver-directed AAV-mediated gene therapy application. Consistent with data reported previously, the ET3 transgene enabled 20-fold higher expression compared to that achieved using the BDD human fVIII transgene in transiently transfected HepG2 cells. Furthermore, the enhanced expression conferred by the ET3 transgene compared to the BDD human fVIII transgene was recapitulated *in vivo* via hydrodynamic plasmid injection, where ET3 achieved 20-fold higher levels of circulating fVIII activity compared to HSQ.

The bioengineered design of ET3 can be compared in some regards to a strategy currently being pursued in AAV clinical gene therapy of hemophilia B where constructs encoding a naturally occurring human coagulation factor IX molecule termed factor IX-Padua that exhibits greater specific procoagulant activity than normal human fIX,^27^ and thus also increase vector potency. In this study, the construct modifications implemented were designed to enhance expression/secretion efficiency, a bioengineering strategy which similarly increases vector potency. However, unlike the factor IX-Padua mutation, which is a naturally occurring mutation, ET3 is a synthetic engineered fVIII incorporating high expression elements derived from endogenous porcine fVIII sequence.^13^

When inserted into the rAAV-HCR-hAAT vector backbone, the expected genome size was 5.9 kb, which substantially exceeds the 4.7 kb size of endogenous AAV genomes. It seems likely that further improvement in both titer and potency could be garnered by reducing the vector genome size similar to what was described previously by Nathwani and colleagues.^23^ Unlike other, smaller, bioengineered vectors, such as that reported by the Nathwani group, molecular analysis of rAAV-HCR-ET3 revealed that there was no detectable full length transgene packaged into the vector.^23^ Rather, consistent with previous reports of oversized vectors, molecular analysis of the rAAV-HCR-ET3 vector revealed that it packaged primarily as truncated transgene fragments, with no detectable viral transgenes larger than 5.0 kb^3,4^ PCR analysis of the purified viral genomes revealed a packaging bias favoring the central portion of the transgene. This result is consistent with the current model of the AAV ssDNA transgene packaging from the 3′ end of both sense and antisense strands, and, in the case of oversized transgenes, being truncated somewhere prior to the 5′ ITR through a presently unknown mechanism. This model predicts that most viral particles will carry either a plus or minus template both of which will contain A2-A3 domain transgene sequences, while the HCR-A1 transgene regions will only be carried by minus strand containing viral particles, and the C1-C2-bGHPA transgene regions carried only by the plus strand containing viral particles. This differential prevalence of transgene region frequency, combined with the sub-5.0 kb smear of viral ssDNA detected by Southern blot analysis, suggests a heterogenous population of partially encoding viral transgenes. Therefore, it is possible that intracellular reassembly of the minus strand viral genomes containing the complete HCR promoter region and truncated fVIII template may lead to the production of dysfunctional fVIII protein products, presenting a potential safety concern by exposing treated individuals to novel immunological epitopes.

Previous work by the Nathwani lab with a similarly sized AAV-fVIII vector failed to detect either persistent truncated fVIII transgene or truncated fVIII protein in mice treated with the oversized vector.^23^ To alternatively investigate the presence of full-length fVIII mRNA in AAV treated mice, we performed RT-PCR analysis of mRNA transcripts (Supplementary Figure S3) in the livers of treated mice. This analysis showed the presence of the C2 domain of fVIII, an area found to be preferentially truncated in the packaged viral transgenes, which is only possible if the complete 5′ sequence upstream of the interrogated 3′ sequence region is present. These findings, coupled with the high specific activity of circulating fVIII in AAV-treated mice (Figure 3f), supports a model wherein, despite the truncated nature of the vector transgene, the majority of translated protein product contains the complete fVIII peptide sequence.

The differential prevalence of viral ssDNA transgene sequences in the product preparations necessitates considerations that must be taken into account when titering oversized rAAV vectors using PCR-based methods. The heterologous nature of the viral particles suggests that the quantity of a single region of ssDNA within the transgene may not be representative of entire viral genome. This brings to light an important safety consideration, as it has been shown in clinical trials that rAAV vector doses greater than 2e12 vg/kg can induce acute liver toxicity due to capsid-targeted cytotoxic T cell-mediated destruction of transduced cells.^6,28,29^ In this study, rAAV-HCR-ET3 was titered by quantitative protein analysis of DNA-containing viral particles. This approach provides an accurate estimation of the number of viral particles that contain DNA in the preparation, but does not account for the regions of transgene DNA that has been packaged. As there were no detectable full-length transgenes packaged within the rAAV capsids, titering oversized rAAV vectors by either determination of viral particle number by mass or optical density, or by use of qPCR amplicons directed to a single viral genome segment, does not describe the heterogeneous nature of the vector genomes in the preparation. This presents a significant challenge to vector product manufacturing, characterization, and quality metrics for clinical product development comprising oversized AAV vector genomes.

Proviral rAAV-HCR-ET3 genomes persisted in the livers of treated mice for the duration of the study (between 11 and 50 weeks) as determined directly by qPCR and indirectly by the continuous detection of circulating fVIII activity. While the vector copy numbers obtained in this study are substantially lower than those seen in previous studies using similar vectors,^22,23^ cellular DNA was collected soon after AAV administration, between 6 and 17 weeks. In this study, vector doses that were comparable to those used in the previous studies were collected as long as 48–50 weeks after vector administration. The low vector copy numbers in this study may be explained by loss of episomal AAV DNA over this extensive time period.

Despite its fragmented packaging, rAAV-HCR-ET3 provided complete (≥ 100% normal) correction of the fVIII deficiency at doses as low as 2e12 vp/kg. Additionally, doses as low as 1e12 vp/kg provided lower, but still curative fVIII activity levels. Finally, a minimally effective dose of 5e11 vg/kg was identified based on the criteria of achieving sustained therapeutic levels of fVIII between 0.02–0.12 units/ml for more than 10 weeks after vector administration. In addition to measurement of plasma fVIII activity, phenotypic correction was demonstrated by hemostatic challenge using the tail clip bleeding assay, which demonstrated dose responsive correction of bleeding diathesis. Although an overall reduction in bleeding was observed that trended toward significance in terms of a quantitative correlation between blood loss and vector dose or plasma fVIII activity level, previous experience with this assay in our laboratory has shown that blood loss does not directly correlate to fVIII activity levels in plasma. Furthermore, as others have shown, it frequently is observed that mice with levels of fVIII activity below the threshold of detection (1.6% of normal) fail to lose detectable amounts of blood. It is possible, therefore, that sub-detectable levels of fVIII activity may be sufficient for the correction of bleeding diathesis in this murine model.

Initial exposure to fVIII by persons with hemophilia A represents an immunologic challenge that can lead to the development of neutralizing humoral immunity in ~25% of persons with severe hemophilia A.^5,30^ Since rAAV gene therapies are designed to provide a continuous source of the exogenous protein in treated individuals, this response presents a considerable safety and efficacy concern as these vectors are translated into human trials. Indeed, when administered to immune-competent macaques, rAAV encoding a codon optimized BDD human fVIII molecule resulted in the formation of neutralizing antibodies to fVIII.^23^ Furthermore, the incidence of anti-fVIII inhibitor formation in persons with hemophilia A is significantly greater than that of anti-factor IX inhibitors in persons with hemophilia B.^5^ Thus, immunogenicity remains among the most significant safety concerns for all new hemophilia A therapeutics including gene therapies.^31,32^ Despite the demonstrated safety in the ongoing clinical trial of scAAV2/8-LP1-hFIXco for the treatment of hemophilia B, these results may not be predictive of safety in a similarly designed clinical trial for AAV-fVIII vectors for the treatment of hemophilia A.^33^ As there has not been a clinical trial of AAV gene therapy for hemophilia A completed to date, this issue can only be discussed at this time using preclinical data. In this study, we utilized the exon-16 disrupted murine model of hemophilia A on predominantly a C57Bl/6 genetic background, which previously was shown to produce higher anti-fVIII inhibitory antibodies than the Balb/c model of hemophilia A.^34^ Using this model, two animals developed inhibitors to ET3, as evidenced by loss of fVIII activity and confirmed by ELISA and Bethesda assays. The two mice that developed anti-fVIII immune responses received the highest doses of virus (2e13 and 1e13 vp/mouse) and exhibited supraphysiological levels of circulating fVIII prior to inhibitor development. This finding suggests that in immune-competent individuals, there may be an upper vector dose limit imposed by both the viral capsid antigen load and the plasma fVIII load (potency). The possibility remains that the unique porcine sequences known to be necessary for high-level expression in ET3 also conferred increased immunogenic risk and were the target of the anti-fVIII immune response observed. However, Lollar and colleagues recently performed a rigorous comparison of BDD rhfVIII and rpfVIII in the murine hemophilia A model, which revealed that the overall immunogenicities were similar with anti-A2 and anti-C2 constituting the majority of the inhibitors detected against each molecule.^35^ An important safety consideration in light of the high expression porcine sequences present in ET3 is that both plasma-derived and now rpfVIII products have been used clinically, the former for more than two decades, to treat acute bleeding following the development of anti-hfVIII inhibitors in the settings of congenital and acquired hemophilia A.^36,37^ Therefore, there may be sufficient data available to support the risk/benefit case for transition to clinical safety (including immunogenicity) testing of ET3 delivered by AAV vector, lentiviral vector or intravenous infusion of the recombinant molecule.

As predicted by all previous gene transfer and recombinant protein expression studies, the ET3 transgene demonstrates high-level transgene product biosynthesis following liver-directed rAAV delivery. The current data directly support the benefit of incorporating the high expression fVIII sequence elements encoded within ET3 into AAV gene transfer systems for treatment of hemophilia A. The rAAV-HCR-ET3 vector provided a long-term source circulating fVIII activity and correction of bleeding diathesis at vector doses lower than those of other bioengineered fVIII variants despite its larger size, making ET3 an ideal candidate transgene for incorporation into smaller AAV vector systems. However, due to the immune response exhibited by some mice against fVIII, additional immune modulation may be necessary to prevent the development of humoral immune response to fVIII. Previous data from our lab has shown that *ex vivo* genetic modification of hematopoietic stem cells (HSC) using a lentivector gene transfer platform promotes immune non-responsiveness to fVIII.^38^ While the immune nonresponsive profile induced by *ex vivo* HSC gene modification is desirable and this approach is predicted to be curative, the overall approach is clinically more complex than a single peripheral vein infusion of rAAV. Furthermore, the preconditioning regiments needed to attain therapeutic levels of cellular engraftment and protein expression pose additional risks. Therefore, it seems logical that different gene therapy strategies may be more amenable than others to application in distinct geographical, economically developed and clinically advanced populations.

## Materials and Methods

### Vector cloning

The previously described HP47/ET3 transgene^13^ was released from the ReNeovector backbone by digesting with NotI followed by Klenow fragment fill-in and subsequent *SpeI* digestion. The previously described AAV2 vector backbone AAV-HCR-hAAT-FIX^15^ was digested with *BglII* followed by Klenow fragment fill in and subsequent *NheI* digest. The AAV-HCR-hAAT vector backbone was ligated to the ET3 transgene, generating the rAAV-HCR-ET3 viral vector expression plasmid. An equivalent AAV expression vector encoding a previously described non-bioengineered BDD hfVIII construct (designated HSQ)^39^ was generated by releasing HSQ with *NotI* followed by Klenow fragment fill in and subsequent *XhoI* digestion. AAV-HCR-hAAT-FIX was digested with *BglII* followed by Klenow fragment fill in and subsequent *SalI* digestion. AAV-HCR-hAAT was ligated to the HSQ transgene, generating the rAAV-HCR-HSQ viral vector expression plasmid.

### Transient transfection

Naive human hepatocellular liver carcinoma (HepG2) cells were grown to 75% confluency in six-well plates. Cells were transfected with 2 µg of rAAV-HCR-ET3 or rAAV-HCR-HSQ viral expression plasmid using 6 µl Lipofectamine 2000 (Life Technologies, Carlsbad, CA) in DMEM supplemented with 10% fetal bovine serum. Twenty-four and forty-eight hours after transfection, media was replaced with 1 ml AIM-V serum free medium (Life Technologies). Seventy-two hours after transfection, conditioned media was collected and analyzed for fVIII activity by one stage clot assay as previously described.^39^

### Vector production

The recombinant AAV8 vector encoding ET3 was produced as described previously.^19^ Briefly, human embryonic kidney (HEK) 293 cells were transfected by calcium phosphate using the following plasmids: (i) the adenovirus helper plasmid AdHP encoding adenovirus helper proteins E2 and E4, (ii) the AAV8 packaging plasmid AAV8PK encoding AAV2 Rep and AAV8 Cap, and (iii) the vector plasmid encoding the transgene expression cassette. The transfected cells were harvested 72 hours after transfection, lysed by homogenization, clarified by centrifugation, and vectors purified by precipitation with 8% polyethylene glycol and two rounds of cesium chloride gradient ultracentrifugation. Purified vectors were dialyzed into phosphate buffered saline (PBS) and supplemented with Pluronic F68 (0.001% final concentration). The empty capsid free vector preparations were quantified following SDS-polyacrylamide gel electrophoresis (PAGE) and silver staining by comparing VP1, 2, and 3 staining intensity via scanning densitometry with an established AAV reference vector.

### Molecular studies

Viral ssDNA was isolated from virus stocks using the QIAmpMinElute Virus Spin Kit (Qiagen, Hilden, Germany) according to the manufacturer’s instructions. Preparations for size analysis were prepared free of carrier RNA. Size analysis was performed by alkaline gel electrophoresis and subsequent Southern blot. DNA was probed using a cocktail of biotinylated probes directed against the A2, C2, and bovine polyadenylation sequences (Supplementary Table S1) and detected using the North2South Chemiluminescent Detection kit (Thermo Fisher, Waltham, MA) according to the manufacturer’s instructions. Sizing analysis was performed by alkaline gel electrophoresis and interpolation of sample sizes from a standard curve generated using single-stranded DNA markers. Cellular DNA was extracted from liver tissue following perfusion for 10 minutes with 0.9% saline solution using the DNeasy Blood and Tissue Kit (Qiagen) according to the manufacturer’s instructions. All qPCR was performed using a StepOne real-time PCR platform (Life Technologies) and analyzed on the device’s software. QPCR quantitation was performed using a standard curve generated from dilutions of rAAV-HCR-ET3 viral expression plasmid. For determination of fVIII mRNA quantity in HepG2 cells transfected with AAV-fVIII expression plasmids, mRNA was extracted from cells using the QiagenRNEasy kit, and quantitative reverse-transcription PCR was performed as previously described.^12^ Quantitation of regions of the viral genome was performed in separate experiments using sets of primers directed against regions that spanned the length of the rAAV-HCR-ET3 transgene (Supplementary Table S2). To control for any differences in primer efficiency, each reaction was quantitated separately against plasmid standard curves generated using each region-specific primer pair (Supplementary Figure S1). Isolated viral ssDNA was added to 2x Power SYBR green PCR master mix (Life Technologies) with final concentrations of 0.3125 µM each of forward and reverse primers to a final volume of 20 µl. The data shown is the mean of two qPCR reactions. As all virus used in this study was generated in a single production run, error bars are not shown to avoid confusion between the inter-assay variability and variability due to independent virus preparations. For determination of liver transgene copy numbers, 40 ng of cellular DNA was used as template in 2x Power SYBR Green PCR master mix with final primer concentrations of 0.3125 µM each. The primer set directed against the A2 domain (Supplementary Table S2) was used for determination of transgene copy number in transduced livers. Quantitation was performed using a standard curve generated from rAAV-HCR-ET3 viral expression plasmid. Forty ng of naive liver genomic DNA was added to each standard curve reaction to mimic the cellular DNA environment of the experimental samples. To determine the presence of full-length mRNA in the livers of treated mice, RNA was extracted from liver tissue using the RNeasy kit (Qiagen) according the manufacturer’s instructions. One µg of RNA was subject to DNA removal and reverse transcribed using the Quantitect reverse transcription kit (Qiagen) according to the manufacturer’s instructions. PCR was performed using the primer set directed against the C2 region of ET3 (Supplementary Table S2).

### Animal studies

All animal studies were performed under the guidelines set by the Emory University Institutional Animal Care and Use Committee. Immune competent, exon 16 disrupted C57BL/6 backcrossed mice were used as a murine model of hemophilia A^40^ and C57BL6 background mice used as healthy control. Hydrodynamic plasmid injections were performed in 4–6 week old hemophilia A mice. For these studies, 10 µg of plasmid was diluted into 2.1 ml Transit-EE hydrodynamic delivery solution (Mirus) and delivered by tail vein injection over the course of 7–10 seconds. Blood plasma was collected 24 hours after injection. AAV administration studies were performed in 8–12 week old mice, which were administered a single tail vein injection of AAV viral vector diluted to a volume of 200 µl in sterile filtered DPBS containing Pluronic F68 (0.001%). For both *in vivo* studies, citrated plasma was collected by retro-orbital bleed into one-tenth volume 3.8% trisodium citrate. fVIII activity was measured using the commercially available COATEST SP fVIII assay (Coatest SP, Diapharma, West Chester, OH) according to the manufacturer’s instructions using a standard curve generated from pooled citrated human plasma (George King, Overland Park, KS). Using this standard curve, the limit of detection for the assay was empirically determined to be 0.015 units (U) of fVIII/ml. Baseline determinations of fVIII activity in untreated hemophilia A mice were determined to be below the limit of detection of the assay. Circulating antibodies against fVIII were detected by enzyme-linked immunosorbent assay (ELISA).^41^ Antibodies to fVIII were captured using immobilized recombinant hfVIII (Kogenate, Bayer, Leverkusen, Germany), bound to microtiter plates, and detected using alkaline phosphatase conjugate of goat anti-mouse IgG as previously described.^39,42^ Absorbance at 405 nm was measured from dilutions of test plasma and plotted against the logarithm of the plasma dilution. The resulting sigmoidal curves were fit to the 4-parameter logistic equation by non-linear regression using the Levenberg-Marquardt algorithm. ELISA titer was defined empirically as a dimensionless value calculated from the reciprocal plasma dilution resulting in an optical density of 0.3 from the fitted curves.^42^ The presence of inhibitory antibodies was confirmed by Bethesda assay, performed as previously described.^43,44^ Bethesda titer was calculated as the average of dilutions of inhibitor found to produce between 25% and 75% residual activity.

### *In vivo* specific activity of rAAV-derived fVIII

Antigen concentrations were determined using a capture ELISA designed to detect the heavy chain of fVIII. Monoclonal antibody (421C) directed towards a porcine A1 domain epitope was immobilized on 96 well high binding polystyrene plates (Corning, Corning, NY) and used to capture fVIII antigen from plasma samples diluted in HBS Tween-80. Samples were then incubated with a biotinylated secondary monoclonal antibody (4C7) directed towards a human A2 domain epitope for 1 hour in blocking buffer containing 1% BSA. Full length fVIII was measured using goat anti-mouse IgG antibody conjugated to alkaline phosphatase and *p*-nitrophenylphosphate. Absorbance at 405 nm was read 30 minutes after the addition of the chromogenic substrate. Antigen concentrations were extrapolated from a standard curve derived from purified fVIII protein serially diluted in E-16 disrupted hemophilia A mouse plasma. Specific activity of mouse plasma samples were determined by plotting antigen concentration against fVIII activity measured by COATEST SP fVIII assay kit. Linear regression analysis was performed, comparing AAV treated mouse plasma samples to the standard curve.

### Tail transection assay

Tail transection bleeding assay was performed as previously described.^43^ Briefly, mice were anesthetized under isoflorane and their tails were warmed for 10 minutes in a 37 °C water bath. Tails then were transected 4 mm from the tip using a scalpel and immediately placed into 14 ml vials containing 0.9% saline solution held at 37 °C. Tails were allowed to bleed freely for 45 minutes, at which time they were removed from the vials. Blood loss during 45 minutes was measured by mass of blood accumulated in the tube and normalized to body weight while controlling for evaporative loss.

## Notes

C.B.D. and H.T.S. are cofounders of Expression Therapeutics and own equity in the company. Expression Therapeutics owns the intellectual property associated with ET3. The terms of this arrangement have been reviewed and approved by Emory University in accordance with its conflict of interest policies. J.F.W. holds patents in the field of AAV gene therapy and is a scientific cofounder of and consultant to Spark Therapeutics.
