# Supplementary material for: Bioengineered coagulation factor VIII enables long-term correction of murine hemophilia A following liver-directed adeno-associated viral vector delivery
Source: Mol Ther Methods Clin Dev. 2014 Aug 6;1:14036–. doi: 10.1038/mtm.2014.36 (PMC4362354; doi:10.1038/mtm.2014.36)
Supplement: Supplementary Figures and Tables [file mtm201436-s1.docx]

**Supplemental Table 1**

| A2 | GAAGATGGGCCAACTAAATCAGATCCGCGGTGCCTGACCCGCTATTACTCTAGTTTC |
| --- | --- |
| C2 | CACCTCCAAGGGAGGAGTAATGCCTGGAGACCTCAGGTGAATAATCCAAAAGAGTG |
| bGHPA | GTGCCTTCCTTGACCCTGGAAGGTGCCACTCCCACTGTCCTTTCCTAATAAAATG |

**Biotinylated probes used for detection of AAV-HCR-ET3 viral genomes**

A cocktail of biotinylated probes was used for detection of AAV-HCR-ET3 viral genomes during Southern blot analysis.

**Supplemental Table 2**

| **Name** | **Sequence** | **Amplicon Length (bases)** |
| --- | --- | --- |
| HCR + | TTCGGTAAGTGCAGTGGAAG |  |
| HCR - | GTCCTCGTCCGTATTTAAGC | 191 |
| Porcine A1 + | CCTGAAGAACATGGCTTCTC |  |
| Porcine A1 - | TACCGGGAAGGACTTTATCG | 133 |
| Human A2 + | CTCACGGAATCACTGATGTC |  |
| Human A2 - | TTGGCCCATCTTCTACAGTC | 136 |
| Porcine A3 + | TGGAGCAGCTCTGGGATTAC |  |
| Porcine A3 - | GCAAATTCCCGGAAGACCAC | 109 |
| Human C1 + | TCAATGCCTGGAGCACCAAG |  |
| Human C1 - | AGATGTAGAGGCTGGAGAAC | 119 |
| Human C2 + | CCTCCAAGGGAGGAGTAATG |  |
| Human C2 - | CATCTTGACTGCTGGAGATG | 169 |
| bGHPA + | CCTTCTAGTTGCCAGCCATC |  |
| bGHPA - | CCAGCATGCCTGCTATTGTC | 199 |

**Primers used for regional transgene analysis**

Primer sets spanning the length of the AAV-HCR-ET3 viral transgene were used for quantitative PCR analysis of the packaged ssDNA content of AAV-HCR-ET3 viral particles.

**Supplemental Figure 1**

**A1**

**HCR**


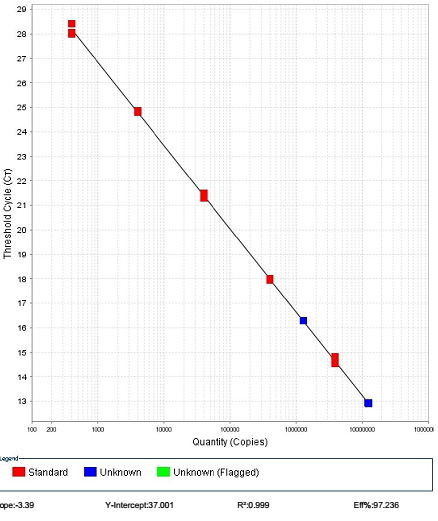

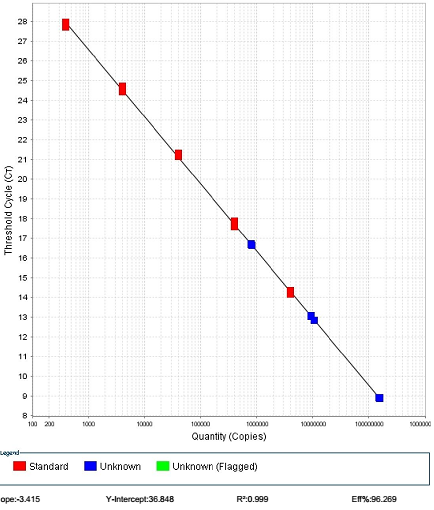


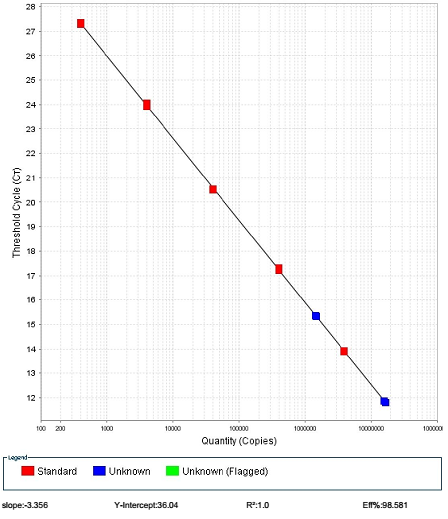

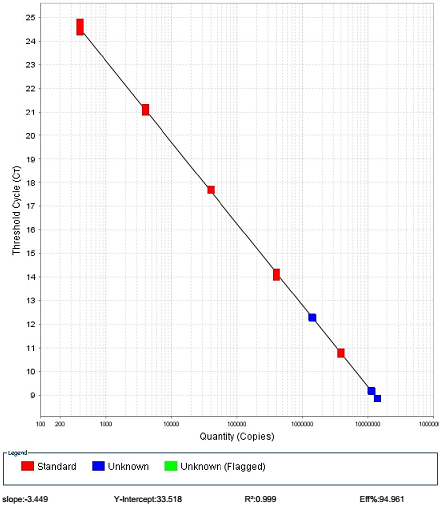


**A2**

**A3**


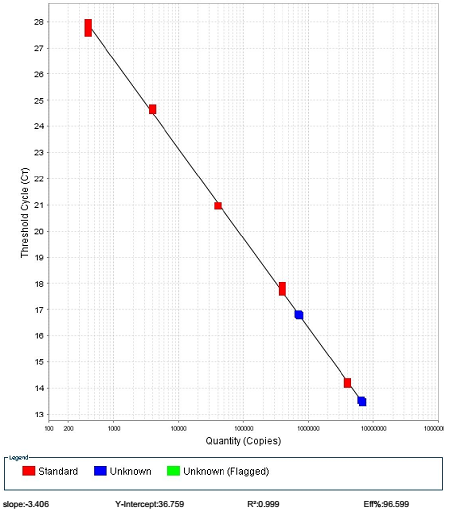

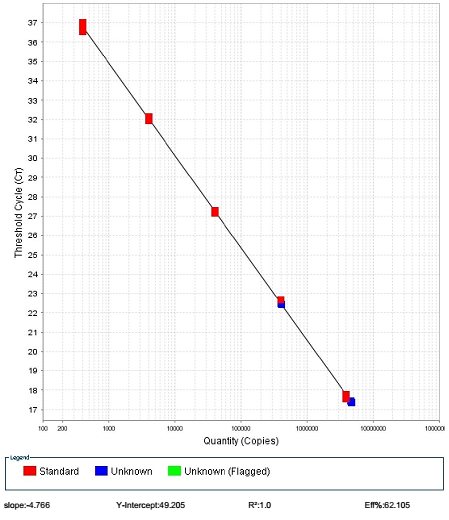

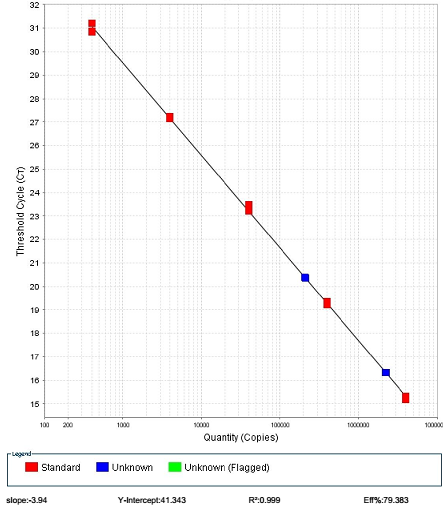
a

**C2**

**C1**

**C2**

**C1**

bGHPA

**bGHPA**


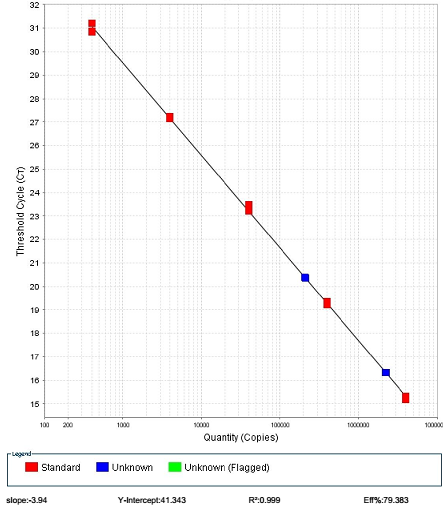


**Standard curved for quantitative PCR analysis**

To control for variation in primer efficiency during quantitative PCR analysis, standard curves of AAV-HCR-ET3 viral expression plasmid were generated for each primer set spanning the length of the AAV-HCR-ET3 transgene.

**Supplemental Figure 2**

**
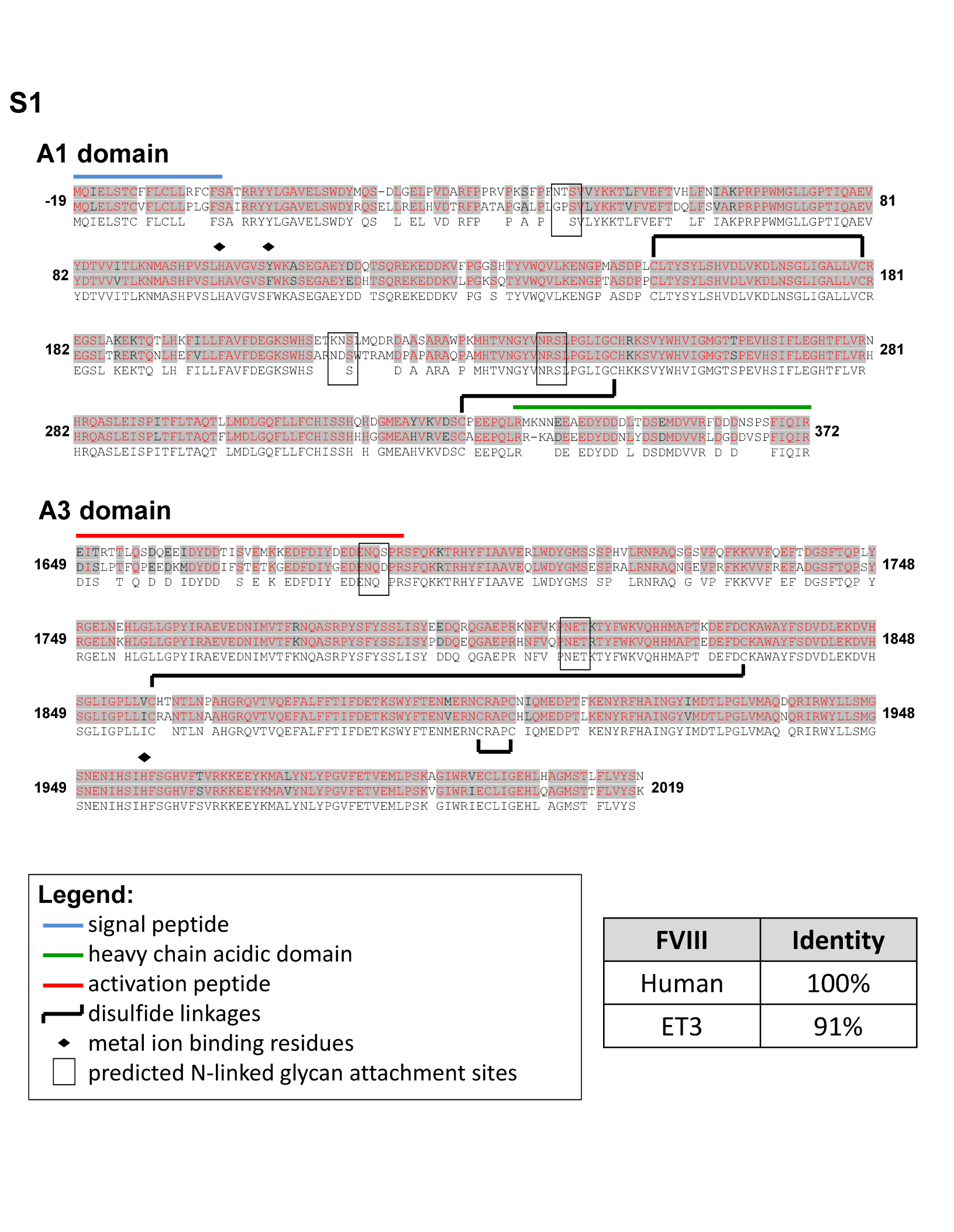
**

**Sequence alignment of ET3 and HSQ**

Amino acid sequence alignments for the signal peptide (black bar), A1 domain, heavy chain acidic domain (green bar), activation peptide (red bar) and A3 domain of human (top) and ET3 (bottom) fVIII are shown. Identical residues are distinguished by red type with gray background, similar residues are shown black type with gray background and all other residues are displayed in black type with transparent background. Disulfide linkages are noted by the black lines connecting cysteine residues. Places where either human, ET3 or both sequences encode an N-linked glycosylation attachment site (N-X-S/T) are outlined with a black box.

**Supplemental figure 3**

**
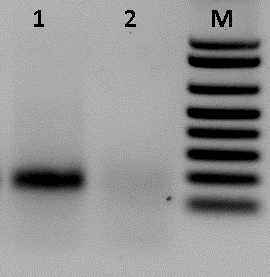
**

**ET3 C2 domain sequence RNA is present in liver of treated mice**

Reverse transcription PCR analysis of RNA isolated from livers of treated and untreated mice shows ET3 C2 domain sequence in a mouse treated with AAV-HCR-ET3 (lane 1) and no detectable ET3 C2 domain sequence in untreated control (lane 2).
